# The effect of nano hydroxyapatite coating implant surfaces on gene expression and osseointegration

**DOI:** 10.4317/medoral.26303

**Published:** 2023-11-22

**Authors:** Hironori Kasai, Edmara TP Bergamo, Ísis de Fátima Balderrama, Kentaro Imamura, Lukasz Witek, Ernesto B Benalcázar Jalkh, Estevam A Bonfante, Kenji Inoue, Paulo G Coelho, Seiichi Yamano

**Affiliations:** 1 Department of Prosthodontics, New York University College of Dentistry, New York, NY, USA; 2 Biomaterials Division, NYU College of Dentistry, New York, NY, USA; 3 Department of Diagnosis and Surgery, School of Dentistry of Araraquara, Sao Paulo State University, Araraquara, Sao Paulo, Brazil; 4 Department of Periodontology, Tokyo Dental College, Tokyo, Japan; 5 Department of Biomedical Engineering, NYU Tandon School of Engineering, New York, NY, USA,; 6 Hansjörg Wyss Department of Plastic Surgery, NYU Grossman School of Medicine, New York, NY, USA; 7 Department of Prosthodontics and Periodontology, Bauru Sc3hool of Dentistry, University of Sao Paulo, Bauru, SP, Brazil; 8 Division of Plastic Surgery, Department of Surgery, University of Miami Miller School of Medicine, Miami, FL, USA; 9 Department of Biochemistry and Molecular Biology, University of Miami Miller School of Medicine, Miami, FL, USA

## Abstract

**Background:**

Hierarchical micro-nano structured topography along with surface chemistry modifications of dental implants have been suggested to positively contribute to the osseointegration process. However, the effect of such surface modifications on the molecular response as well as bone formation rate and quality are still unclear, especially in the early healing period. This study aimed to evaluate the effect of coating a double acid etched (DAE) implant surface with nano-sized (20 nm) hydroxyapatite (Nano) with respect to gene expression, histologic parameters, and nanomechanical properties when compared to DAE control at 1 and 2 weeks after implant placement in a rodent femur model.

**Material and Methods:**

Expression of bone-related genes was determined by qRT-PCR (Col-I, Runx-2, Osx, Opn, Ocn, Alp). Histomorphometric evaluation of bone-to-implant contact (BIC) and bone area fraction occupancy (BAFO) within implant threads was performed using photomicrographs after histologic processing. Mechanical properties, reduced elastic modulus and hardness, were determined through nanoindentation.

**Results:**

At 1 week, the nano group demonstrated significantly higher expression of Col-I and Ocn compared to the DAE group, indicating upregulation of osteoprogenitor and osteoblast differentiation genes. At 2 weeks, Nano surface further exhibited enhanced gene expression of Col-I and Osx in comparison to the DAE surface, suggesting an increased mineralization of the newly formed bone. Nanoindentation analysis revealed that the Nano group presented no significant difference on the ranks of reduced elastic modulus and hardness compared to DAE for both timepoints. Histomorphometric analysis yielded no significant difference in the percentage of BIC and BAFO between the Nano and DAE surfaces at 1 and 2 weeks. However, Nano implants did present a higher mean value, ~50%, of BIC compared to DAE, ~30%, after 2 weeks *in vivo*.

**Conclusions:**

While no significant differences were observed in the amount and mechanical properties of newly formed bone, Nano surface positively and significantly increased the expression osteogenic genes compared to DAE surface at early healing periods.

** Key words:**Dental implants, osseointegration, gene expression, histology, mechanical properties.

## Introduction

From an engineering perspective, microstructured surfaces have first shown to maximize osseoconduction around dental implant by increasing the available surface area for blood clot adherence and protein adsorption, modulating the host response when compared to conventional machined surfaces (1,2). An approach to further increase the biomimicry and bioactivity of endosteal implants has been the nanoscale and/or chemistry modifications of the surface, where increased molecular interactions enhanced selective protein adsorption, further modulating osseointegration phenomena (3-5).

In this context, the incorporation of calcium phosphate (CaP), such as hydroxyapatite, on the implant surface have shown to facilitate bone healing response due to the intrinsic chemistry properties of CaP, which is chemically analogous to the bone; and stimulates cellular migration and formation of bone towards the implant surface (6,7). However, there have been previous reports of adverse biological events associated with hydroxyapatite coatings (8-10). Particularly, the CaP layer thickness has shown to play a critical role in the healing response, with “thick” plasma-sprayed hydroxyapatite coatings being more prone to delamination and to inflammation resulting from the release of apatite particles, which may increase the rate of clinical complications and implant failure (8-10). In an effort to improve hydroxyapatite coating properties and minimize adverse effects, different coating protocols have been explored(6,11), such as resorbable bioceramic media grit blasting and wet chemical methods (12,13).

Implant surface chemistry and texture modifications at the nanoscale level have previously shown to significantly facilitate osseointegration due to increased interaction at the biomolecular level and modulation of cellular response (e.g., apoptosis, differentiation, and growth) (14). Implants with nanotopography have demonstrated enhanced differentiation of mesenchymal and pre-osteoblast cells *in vitro*, which was suggested by significantly higher levels of gene expression of human mesenchymal stem cells, and osteocalcin (Ocn), and osteoprotegerin (Opg), respectively (15). In agreement with *in vitro* data, previous animal studies investigating histological or biomechanical parameters have suggested that the presence of nanostructured implant surfaces improveS the amount of bone formation (16,17), with an up-regulation of osteogenic gene expression (4,5), which was further enhanced by nanocoating with hydroxyapatite (4).

The present study aimed to further investigate the effect of coating a double acid etched (DAE) implant surface with nano-sized (20 nm) hydroxyapatite (Nano) on gene expression, histologic parameters, and nanomechanical properties compared to the conventional, gold-standard, DAE surface at early healing response, 1 and 2 weeks after implant placement, in a rodent femur model. The postulated null hypothesis was that the type of implant surface would not influence gene expression and bone healing and quality parameters.

## Material and Methods

Dental implants (2.7 mm in length by 1.4 mm in diameter) with 2 types of surface treatment were provided by the manufacturer (S.I.N. Implant System, Sao Paulo, SP, Brazil): a dual acid etched surface (DAE) and a dual acid etched coated with a 20nm-thickness layer of hydroxyapatite surface (Nano - Promimic HAnano™ method) (*n*=20 implants/each), both which have been previously characterized (13,16).

Animal experiments were approved by New York University’s Institutional Animal Care and Use Committee (IACUC) (protocol #160207-01). The present study used a Sprague-Dawley male rat femur preclinical animal model (n = 20; 8-10 weeks old, 300-350 g each).

The surgical areas, flat medial surface of the femur of both sides, were shaved and washed with 70% ethanol before surgery. Then, general anesthesia was first induced via intramuscular injection with 0.3-0.4 ml of ketamine/xylazine (80-100 mg/kg and 10-20 mg/kg body weight, respectively). After anesthesia induction, the surgical area was exposed with an incision on the medial surface of the femur using a blade. Implants were placed in osteotomy beds prepared using a No.4 round burr (1.4mm diameter) under saline irrigation to the level of the first layer of cortical bone. After surgery, the tissues were closed by layers using 5-0 absorbable sutures (Henry Schein, Melville, NY). Post-surgery, buprenorphine (0.01-0.05 mg/kg) was administered to control pain for 3 days. The implants remained for 1 or 2 weeks *in vivo* since main effect of surface treatment modifications occur at early healing timepoints (18). After euthanasia by anesthesia overdose, the implants and surrounding bone tissue were retrieved and prepared for gene expression, histological, and mechanical characterizations.

For qRT-PCR, the retrieved samples containing the implants were placed into Trizol reagent (Invitrogen, Carlsbad, CA) at -80°C. RNA was extracted from the pulverized bone powder using Trizol reagent according to the manufacture’s protocol. Then, RNA levels were measured using a NanoDrop ND-2000 Spectrophotometer (NanoDrop Technologies, Wilmington, DE) and treated with DNase I. Target-specific PCR primers for type I collagen (Col-I), runt-related transcription factor 2 (Runx-2), osterix (Osx), osteopontin (Opn), osteocalcin (Ocn), alkaline phosphatase (Alp) and β-actin (as for an internal control) were designed using the ProbeFinder assay design software. cDNA was synthesized using a total of 1 µg RNA using QuantiTect® Quantiscript reverse-transcriptase and RT Primer Mix (Qiagen, Valencia, CA), according to the manufacturer’s protocol. Reactions for the Chromo4 (Bio-Rad, Hercules, CA) were performed in 20-µl reaction volumes for the genes encoding Col-I, Runx-2, Osx, Opn, Ocn, Alp and β-actin using 100 ng of cDNA under the following conditions: 95ºC for 5 minutes, 50 cycles for 95ºC for 10 seconds, 60ºC for 15 seconds, and 72ºC for 1 second. To confirm qRT-PCR specificity, gel electrophoretic assessment was conducted. Each product size of PCR was the following (unit bp): Col-I, 106; Runx-2, 150; Osx, 166; Opn, 216; Ocn, 354; Alp, 183; β-actin, 150. The method used for obtaining quantitative data of relative gene expression was the comparative Ct method (also known as the 2-Ct method). All the results were normalized to β-actin gene. All the graphic data for mRNA expression are presented as the fold expression relative to the control group. Mean values of the triplicates were used for statistical analysis.

For histology and nanoindentation, the bone samples were stored in 70% ethanol (EtOH) for 24 hours and subsequently subjected to progressive dehydration through a series of alcohol solutions (70% to 100% ethanol) and embedded in a methyl methacrylate, according to the manufacturer's instructions (Technovit 9100, Heraeus Kulzer GmbH, Wehrheim, Germany). The blocks were sectioned following the long axis of the implants with a precision diamond saw (Isomet 2000, Buehler, Lake Bluff, IL) and glued to an acrylic slide. Then, samples were grinded (400-2400 grit SiC abrasive papers) and polished (diamond suspension solutions of 9-1 μm particle size; Isomet 2000, Buehler, Lake Bluff, IL) using a grinding/polishing machine (Metaserv 3000, Buehler) under irrigation to a final thickness of approximately 50 μm. Two groups of implant slides were created: one for nanoindentation and one for non-decalcified histology.

Histological images were scanned using a light microscope (Leica DM2500M, Leica Microsystems GmbH, Wetzlar, Germany) and a computer software (Leica Application Suite, LeicaMicrosystems GmbH). The bone-to-implant contact (BIC) along the implant and the bone area fraction occupancy (BAFO) within the implant thread chambers were calculated Using imageJ (National Institute of Health) software by a single examiner blinded to experimental groups.

Nanoindentation testing was utilized to evaluate the mechanical properties of the bone tissue. Indentation (n = 30/specimen) was performed with a nanoindenter (Hysitron TI 950, Minneapolis, MN, USA) equipped with a Berkovich diamond three-sided pyramid probe (16,19). A loading profile with a peak load of 300 μN at a rate of 60 μN/sec, followed by a dwell time of 10 sec and an unloading time of 2 sec was utilized. The delayed dwell time permits the bone to stabilize, in an effort to circumvent any potential creep effect. Each individual implant slide had its mechanical testing performed within the threads of the implant, with each indentation yielding its own respective load-displacement curve (16,19). From the respective curves, the elastic modulus (GPa) and hardness (GPa) of the tested bone tissue were calculated (19).

For gene expression analysis, an ANOVA and Tukey tests were performed to evaluate differences between the groups using SPSS (IBM SPSS 23, IBM Corp., Armonk, NY, USA) (α=0.05). For both the histological and nanomechanical testing, a general linear mixed model and Tukey tests for multiple comparisons were performed to determine differences between the groups using SPSS (α=0.05). The nanomechanical testing results and inferences which are presented are based on ranked data.

## Results

**Figure 1:**
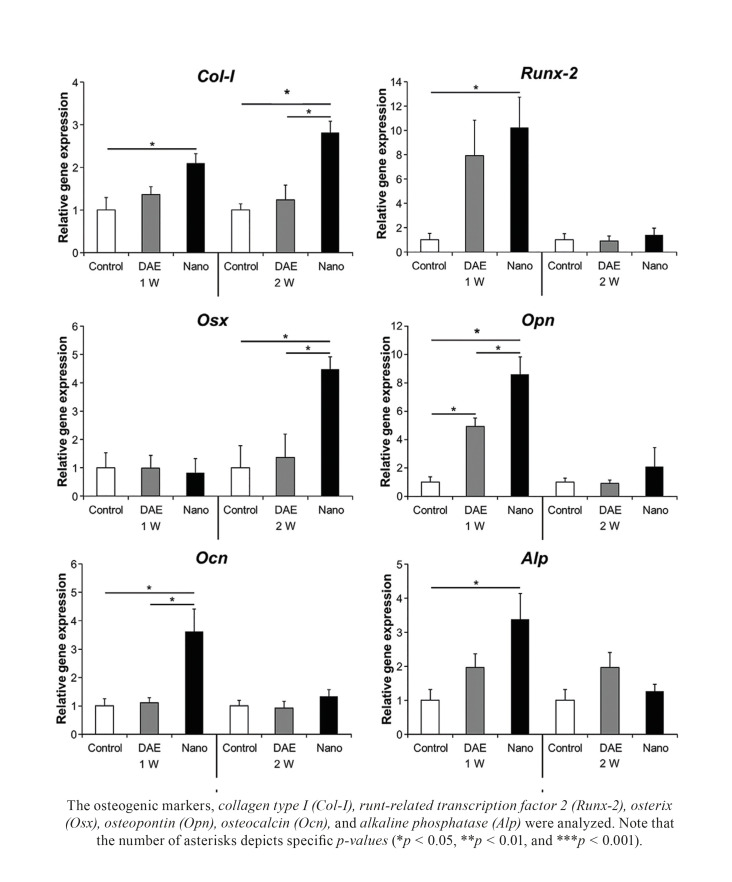
Gene expression levels of selected markers quantified by qRT-PCR.

At 1 week, Nano displayed significantly higher gene expression of Col-I (2.1-fold), Runx-2 (10.2-fold), Opn (8.6-fold), Ocn (3.6-fold), and Alp (3.4-fold) compared to control (*p* < 0.05). DAE induced significantly higher gene expression of Opn (4.9-fold) than control (*p* < 0.05). In addition, Nano exhibited levels of Opn (1.7-fold) and Ocn (3.3-fold) over those seen in DAE (*p* < 0.05). At 2 weeks, the expression level of Col-I (2.8-fold) and Osx (4.5-fold) in Nano was significantly higher than control (*p* < 0.05). Moreover, Nano significantly enhanced gene expression of Col-I (2.3-fold) and Osx (3.3-fold) in comparison to DAE (*p* < 0.05) (Fig. 1).

**Figure 2:**
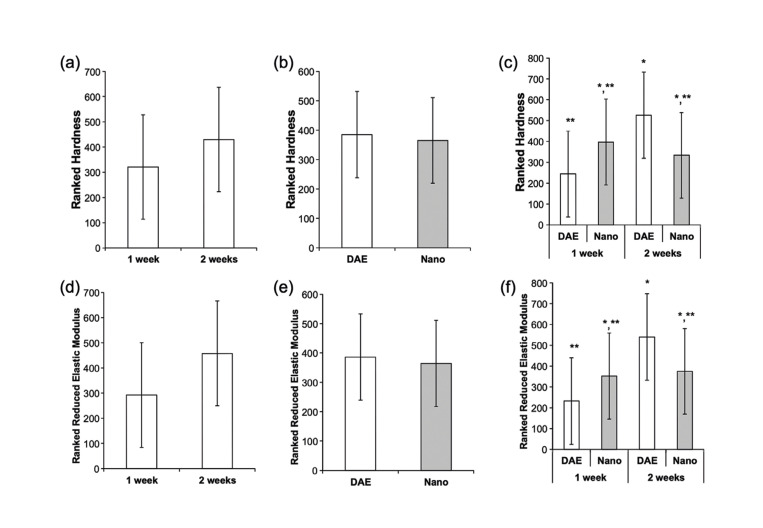
Statistical results summary (mean ± 95%CI) of rank hardness values (a, b and c) and ranked elastic modulus (d, e, and f) with respect to implant surface and time *in vivo*: (a and d) time <italic>in vivo</italic>; (b and e) implant surface; (c and f) time <italic>in vivo</italic> and implant surface. Different symbols indicate statistically significant difference.

When evaluating ranks of hardness and reduced elastic modulus as a function of time *in vivo* (data collapsed over implant surface), no significant difference was detected between 1 and 2 weeks (Fig. 2) (*p*>0.194).

Similarly, when considering implant surface type as a factor (data collapsed over time), no significant difference in hardness or reduced elastic modulus between surfaces was observed (Fig. 2) (*p*>0.106). When surface and time *in vivo* were evaluated concurrently, the Nano surface showed no significant difference in hardness and reduced elastic modulus compared to DAE surface for both timepoints (*p*>0.05). While DAE surface exhibited a significant increase between 1 and 2 weeks in hardness and reduced elastic modulus (*p*<0.05), no significant difference was observed for the Nano surface (Fig. 2) (*p*=0.584).

**Figure 3:**
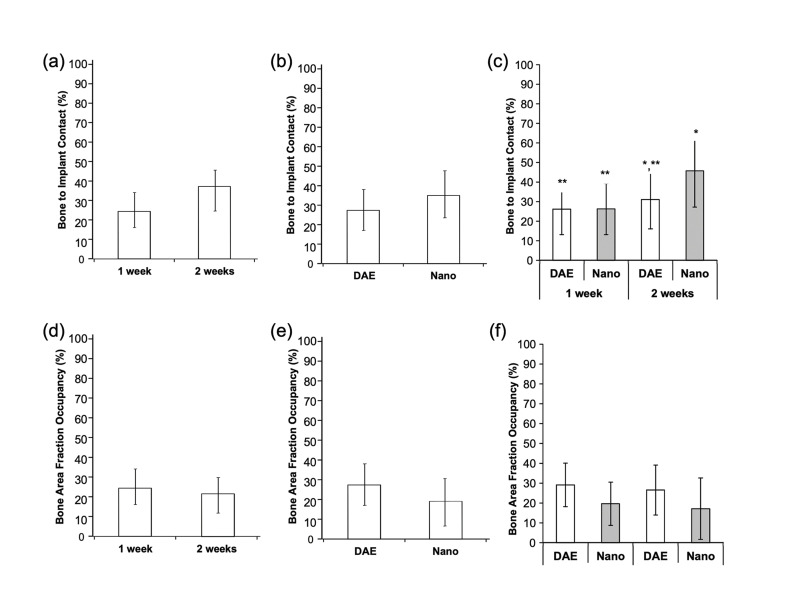
Statistical summary (mean ± 95%CI) of (a, b and c) BIC and (d, e, and f) BAFO with respect to implant surface and time <italic>in vivo</italic>: (a and d) time *in vivo*; (b and e) implant surface; (c and f) time <italic>in vivo</italic> and implant surface. Different symbols indicate statistically significant difference.

The histomorphometric data as a function of time *in vivo* (data collapsed over implant surface) yielded 12% higher percent of BIC at 2 weeks relative to 1 week, although no significant difference was detected between timepoints (Fig. 3) (*p*=0.099). Similarly, no significant difference was obtained between 1 and 2 weeks for BAFO (Fig. 3) (*p*=0.660). When considering implant surface type as a factor (data collapsed over time), no significant difference was observed between Nano relative to DAE for percent of BIC (Fig. 3) (*p*=0.301).

Although 20% higher percent of BAFO for DAE relative to Nano, no significant difference was observed between groups (Fig. 3) (*p*=0.125). Evaluating implant surface and time *in vivo* concurrently, the Nano surface yielded a higher mean BIC value relative to DAE at 2 weeks, though no significant differences were observed between surfaces at both 2- and 1-week timepoints (*p*>0.201). While DAE surface presented no significant increase in the percent BIC when 1 week was compared to 2 weeks timepoints (*p*=0.583), a significant increase was observed for Nano surface at 2 weeks relative to 1 week (Fig. 3) (*p*<0.05). For BAFO parameter, although DAE surface presented a higher tendency to increase bone formation within threads relative to the Nano surface, no significant difference was observed for any pairwise comparisons (Fig. 3) (*p*>0.196).

**Figure 4:**
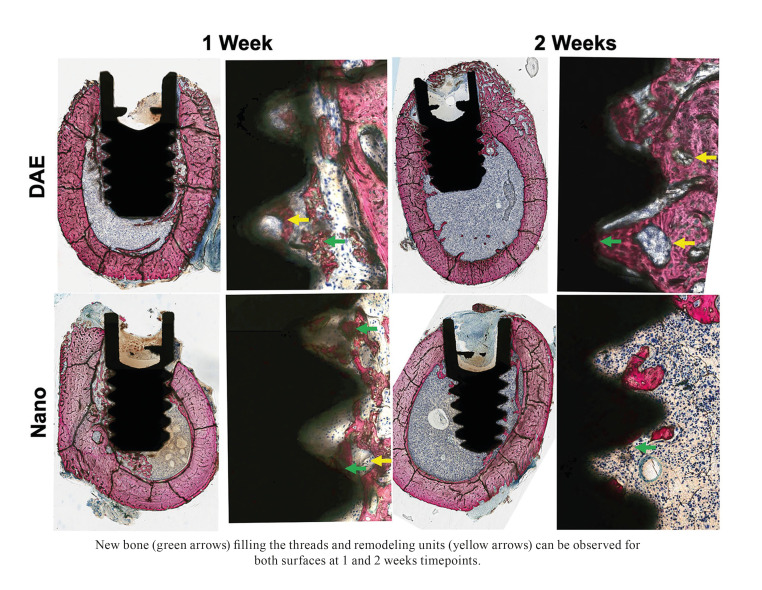
Histological micrographs of Nano and DAE implants.

The histological micrographs revealed newly formed bone with the presence of remodeling units for both groups in contact and in proximity with the implant surface after 1 and 2 weeks of healing, with no visible differences in the amount or type of bone between the implant surfaces (Fig. 4).

## Discussion

Osseointegration of implants is characterized as a structural and functional union bone and implant surface, which ultimately become secondary stability (20). Conventional methods for determining the extent of bone formation around the implant surface depend on molecular analyses, histology/histomorphometry and biomechanics (4,13,16). Therefore, this study investigated the gene expression and characterized the histological and nanomechanical properties of bone formed around a dual acid etched in addition to the nano coating of hydroxyapatite (Nano) surface when compared to the conventional dual acid etched (DAE) surface in a rodent model to recognize the underlying molecular processes and correlate to the observed histological and nanomechanical results. Nano surface positively and significantly increased the expression of osteogenic genes compared to DAE surface, while no significant difference was observed in the amount and mechanical properties of newly formed bone in the early phase of bone healing. Hence, the postulated null hypothesis that the type of implant surface would not influence gene expression and bone healing and quality parameters was rejected.

While the microtopography of implant surfaces has been proposed to act at the cellular level of osseointegration, a surface nanotopography is thought to influence cell-implant interactions at the cellular and molecular level (3-5). It was not until recently that biomedical engineers shifted their attention and focused onto the nanoscale level of implant surfaces (3). Such structurally complex surfaces maximize selective protein adsorption and improve blood clot adherence due to increased surface energy, enhancing platelets activation and producing density gradients of cytokines and growth factors through which a more consistent presence of leukocytes and osteogenic cells results in significantly hastened and higher degrees of osseointegration (1,21). The favorable properties of nanoscale implant surfaces are not merely a result of changes in surface texture but to a large extent due to alterations to the surface chemistry, for example in the current surface through the incorporation of calcium phosphate (CaP) coating on the surface (20nm-thickness hydroxyapatite coating) (13,16). Thus, changes in nano implant surfaces convey their effects at a physical, chemical, and biological level (22).

Hydroxyapatite exhibits functionality in promoting osteoblast adhesion, migration, differentiation, and proliferation; all of which are essential for bone regenerating (6,7). Hydroxyapatite also has the ability to bond directly onto bone due to their similar chemical and structural composition, which has made this ceramic the first-choice for implant applications. The novel modes of application of hydroxyapatite on implants surfaces have resulted in much thinner layers (12,13), herein 20 nm thickness, than those used previously when plasma was sprayed with a minimum coating of 50 μm thickness (8-10). The modern hydroxyapatite applications of nanometer thickness have reduced the risk of biological complications around the implants (23).

There is a substantial body of evidence showing that nantopographic and chemistry surface modifications of implants are associated with the up-regulation of osteogenesis-related gene expression at early stages of bone healing (4,24). Strontium-incorporated titanium oxide surfaces have shown to remarkably up-regulate expression of Runx-2, Osx, bone sialoprotein, and Ocn at 2 weeks of healing (24). In a previous study, the current group has also demonstrated that resorbable bioceramic media grit blasted surfaces presenting nanometer-scale texture within a micrometer-scale texture significantly increased the expression of osteogenic genes compared to conventional micrometer-scale texture DAE surface at early healing periods (4). Similarly, the present study data showed that gene expression levels of osteogenic markers significantly increased for implant surfaces with nano coating of hydroxyapatite (Nano) at 1 and 2 weeks compared to standard DAE. At 1 week, the tissue surrounding the Nano peri-implant surface exhibited significantly higher expression of Col-I, Runx-2, Opn, Ocn, and Alp relative to the tissue around the control surface and higher expression of Opn and Ocn genes than DAE, which indicate superior osteoprogenitor activity (4). At 2 weeks, the expression levels of Col-I and Osx were significantly higher for Nano compared to control and DAE surface, also suggesting increased osteoblast differentiation (25). Runx-2 is a vital transcription factor in osteoblast differentiation (26) and is located upstream to Osx (27), Opn, and Ocn (28). Alp is known to be a regulatory factor for matrix mineralization and is expressed at the early stages of osteogenesis (29). Altogether, nanostructured hydroxyapatite coated surfaces demonstrated increased expression of markers related to early bone formation, growth, and maturation when compared to conventional dual acid etched surfaces.

Literature findings evaluating bone formation and architecture as well as bone quality through histomorphometric analyses and nanomechanical properties, respectively, have reported a higher formation rate and more mature bone architecture surrounding implants with complex nanostructured surfaces relative to grit blasted and/or acid etched surfaces, and both improved over standard machined surfaces (2,4,30). Percent BIC has shown up to 15% increase when hydroxyapatite coated surfaces were compared to grit blasted and/or acid etched surfaces (2,4,30). Similarly, percent BAFO has depicted up to 10% higher values at early healing timepoints for topographically complex surfaces relative to others (2,4). Although no significant difference was observed in the current study between Nano and DAE surfaces for either BIC or BAFO parameters, implants with Nano surface did present 30% higher mean percent of BIC, ~50%, compared to DAE, ~30%, after 2 weeks *in vivo*, which is in line with the cited literature findings.

Although implants with a Nano surface presented higher rank hardness and ranks elastic modulus compared to DAE at the very early time point, no significant difference was observed between them for both timepoints evaluated (1 and 2 weeks *in vivo*). Such data is in contrast with the main body of the literature that has presented an increased degree of mineralization for newly bone formed around implants with nanostructured hydroxyapatite coating (16). The rationale behind the beneficial results of nano hydroxyapatite coating has been associated with the effect of the surface topography and increased biomolecular interactions at the nanoscale level as well as chemistry osseoconductive properties of hydroxyapatite, hastening bone formation as mentioned above, as well as a result of the release of calcium and the phosphate from the surface that might be incorporated into the surrounding new bone and due to the increase in the expression of Alp, strengthening the mineralization process (5,16). However, more experiments must be performed to establish the ideal topographical dimensions and chemistry modifications to understand the mechanisms behind the difference in bone response with different healing periods. Also, large animal models should be planned to obtain more clinically translational data regarding the effect of implant surface modifications on osseointegration.

## Conclusions

Although no significant difference was observed in the amount and mechanical properties of newly formed bone, nanohydroxyapatite coating over a dual acid etched (DAE) surface positively and significantly increased the expression osteogenic genes compared to DAE surface at early healing periods.
